# Prolonged dual antiplatelet therapy for Chinese ACS patients undergoing emergency PCI with drug-eluting stents: Benefits and risks

**DOI:** 10.3389/fcvm.2023.1080673

**Published:** 2023-02-09

**Authors:** Yong Zhang, Chao Chu, Zhong Zhong, Yong-bai Luo, Fei-Fei Ning, Ning Guo

**Affiliations:** ^1^Department of Cardiovascular Medicine, The First Affiliated Hospital of Xi'an Jiaotong University, Xi'an, Shaanxi, China; ^2^Department of Cardiovascular Medicine, Weinan Central Hospital, Weinan, Shaanxi, China

**Keywords:** dual antiplatelet therapy (DAPT), acute coronary syndrome (ACS), major adverse cardiovascular and cerebrovascular event (MACCE), composite bleeding event, emergency percutaneous coronary intervention (PCI)

## Abstract

**Background:**

In patients with acute coronary syndrome (ACS), prolonged dual antiplatelet therapy (DAPT) may reduce ischemic events and increase the risks of bleeding events differently in different ethnic groups. However, whether prolonged DAPT in Chinese patients with ACS following emergency percutaneous coronary intervention (PCI) with drug-eluting stents (DES) will be beneficial or dangerous remains unclear. This study aimed to examine the potential benefits and risks of prolonged DAPT in Chinese patients with ACS who have undergone emergency PCI with DES.

**Methods:**

This study included 2,249 patients with ACS who underwent emergency PCI. If DAPT was continued for 12 or 12–24 months, it was classified as the standard (*n* = 1,011) or prolonged (*n* = 1,238) DAPT group, respectively. The incidence of the following endpoint events was determined and compared between the two groups: composite bleeding event (BARC 1 or 2 types of bleeding and BARC 3 or 5 types of bleeding) and major adverse cardiovascular and cerebrovascular events (MACCEs) [ischemia-driven revascularization, non-fatal ischemia stroke, non-fatal myocardial infarction (MI), cardiac death, and all-cause death].

**Results:**

After a median period of 47 months of follow-up [47 (40, 54)], the rate of composite bleeding events was 13.2% (*n* = 163) in the prolonged DAPT group and 7.9% (*n* = 80) in the standard DAPT group [odds ratio (OR) 1.765, 95% confidence interval (CI) 1.332–2.338, *p* < 0.001]. The rate of MACCEs was 11.1% (*n* = 138) in the prolonged DAPT group and 13.2% (*n* = 133) in the standard DAPT group (OR 0.828, 95% CI 0.642–1.068, *p* = 0.146). The DAPT duration was further shown to be insignificantly correlated with MACCEs as per the multivariable Cox regression model (HR, 0.813; 95% CI, 0.638–1.036; *p* = 0.094). No statistically significant difference was observed between the two groups. However, the DAPT duration was a separate predictor of composite bleeding events according to the multivariable Cox regression model (HR 1.704, 95% CI 1.302–2.232, *p* < 0.001). Compared with the standard DAPT group, the prolonged DAPT group had substantially more BARC 3 or 5 types of bleeding events (3.0 vs. 0.9% in those with standard DAPT, OR 3.430, 95% CI 1.648–7.141, *p* < 0.001) and BARC 1 or 2 types of bleeding events (10.2 vs. 7.0% in those with standard DAPT, OR 1.500, 95% CI 1.107–2.032, *p* = 0.008).

**Conclusion:**

The prolonged DAPT group had a considerably greater incidence of composite bleeding events than the standard DAPT group. No statistically significant difference was observed in the incidence of MACCEs between the two groups.

## 1. Introduction

The most severe form of atherosclerotic cardiovascular disease—acute coronary syndrome (ACS)—is responsible for the majority of cardiovascular disease-related morbidity and mortality worldwide (1, 2). Patients who have experienced an ACS event in the past are at a higher risk for readmission and further severe adverse cardiac events (3). Dual antiplatelet therapy (DAPT) and percutaneous coronary intervention (PCI) have been proven to be effective clinical treatments for patients with ACS (4, 5). To lower the risk of ischemic events, such as stent thrombosis (ST) and recurrent myocardial infarction (MI), recent guidelines in Europe and the United States recommend DAPT with aspirin and a P2Y_12_ inhibitor (clopidogrel, prasugrel, and ticagrelor) for up to 12 months (6–8). After surviving an ACS event, patients still face a high risk of recurrent ischemic events. Studies of patients with ACS from the UK and Belgium reported that 20% of patients died within five years post-ACS, with 13% of those deaths attributed to cardiovascular causes. These findings underscore the need for additional secondary prevention measures beyond the first year of treatment (9). DAPT may be a viable option for lowering the risk level in patients with ACS after one year. The risk of long-term ST and cardiovascular events can theoretically be decreased with prolonged DAPT; however, it will always result in higher risks of bleeding events (10). Whether the administration of DAPT for 12 months allowed patients in certain patient categories to lower their risk of ST or atherothrombotic consequences associated with sites outside the stented segment remains controversial (10–14). While some trials have confirmed its benefit (10, 11), others have not (12–14). According to a previous study, East Asians may have a similar or even reduced risk of developing post-PCI ischemic attacks than Westerners (15). A total of 15,603 patients with atherothrombosis, including 775 Asians, were enrolled in the CHARISMA research (a median follow-up period of 28 months). Asians are more likely to experience moderate Global Utilization of Streptokinase and Tissue-Plasminogen Activator for Occluded Coronary Arteries (GUSTO) bleeding than other races. They also have a lower rate of the composite of cardiovascular death, MI, and stroke during antiplatelet therapy (16). Prolonged DAPT in patients with ACS may reduce ischemic events and increase the risk of bleeding events differently in different ethnic groups. However, the effectiveness and safety of prolonged DAPT in Chinese patients with ACS after emergency PCI with drug-eluting stents (DES) are unknown. In this study, we explored the benefits and risks of prolonged DAPT in Chinese patients with ACS after emergency PCI with DES.

## 2. Methods

### 2.1. Study population

**Figure 1:**
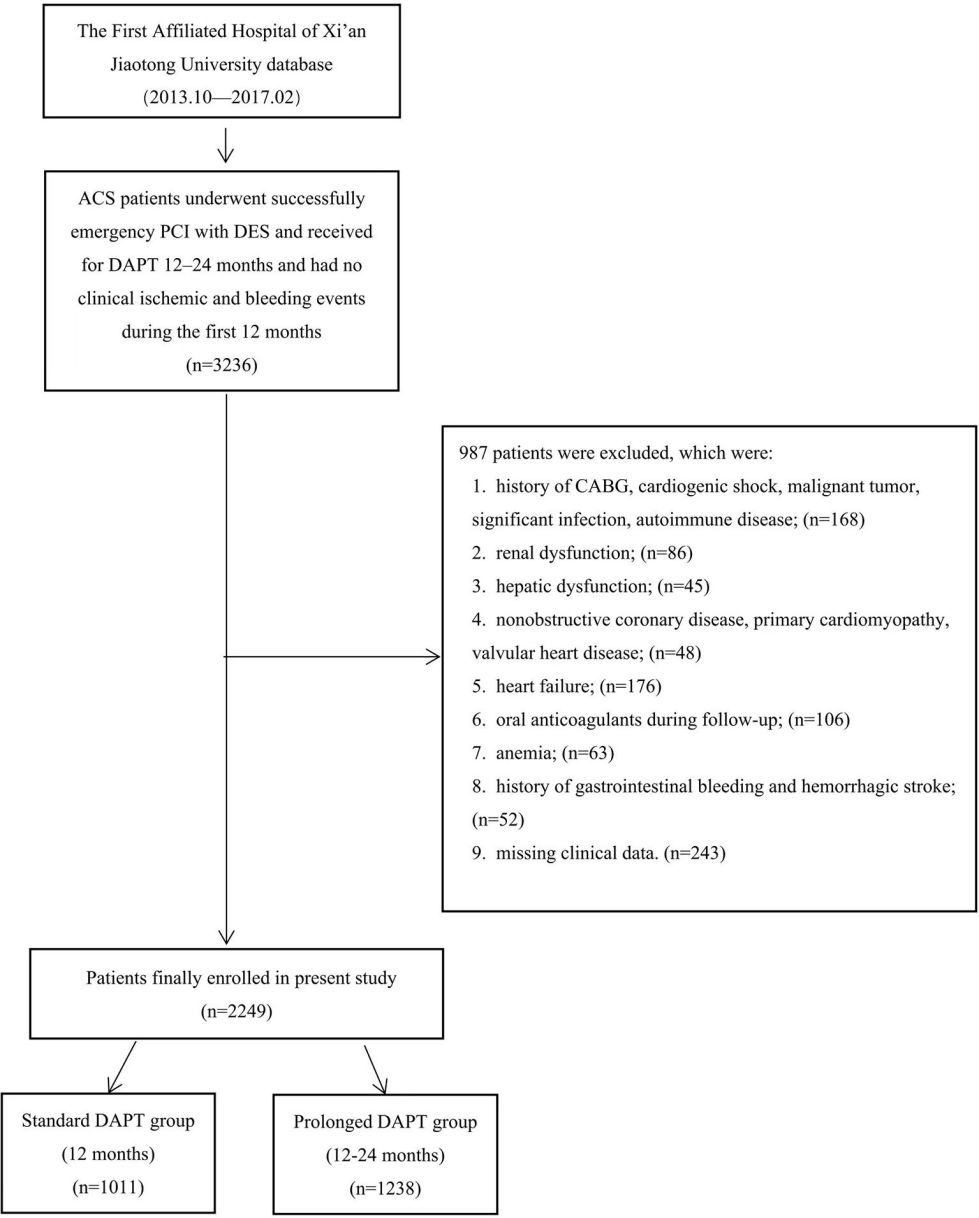
The flowchart of study subject enrollment. ACS, acute coronary syndrome; PCI, percutaneous coronary intervention; DES, drug-eluting stent; CABG, coronary artery bypass grafting; DAPT, dual antiplatelet therapy.

The current analysis is an observational, retrospective cohort study conducted at a single location between October 2013 and February 2017 on patients with ACS who underwent emergency PCI with DES at the First Affiliated Hospital of Xi'an Jiaotong University, Yanta. The inclusion criteria of the current analysis are as follows: (1) patients between the age of 18 and 80 years; (2) patients with ACS who received DAPT for 12–24 months and who had no clinical ischemic or bleeding events during the first 12 months; and (3) patients who successfully underwent emergency PCI with DES. A total of 3,236 patients with ACS were investigated. The exclusion criteria for this study are as follows: (1) a history of coronary artery bypass grafting, cardiogenic shock, malignant tumor, significant infection, or autoimmune disease; (2) a renal disorder with an estimated glomerular filtration rate (eGFR) of < 30 mL/min/1.73 m^2^) or accepted renal replacement treatment; (3) hepatic dysfunction with aspartate transaminase or alanine transaminase levels greater than five upper limits of normal; (4) non-obstructive coronary disease, primary cardiomyopathy, and valvular heart disease; (5) heart failure with left ventricular ejection fraction (LVEF) <30%; (6) oral anticoagulants during follow-up; (7) anemia with hemoglobin (Hb) <60 g/L; (8) a history of gastrointestinal bleeding and hemorrhagic stroke; and (9) missing clinical data. A total of 987 patients were excluded following the exclusion criteria. Finally, 2,249 patients were included in the group. If DAPT was continued for 12 or 12–24 months, it was classified as the standard (*n* = 1,011) or prolonged (*n* = 1,238) DAPT group. The duration of the prolonged DAPT group is 22 (20, 24) months (Figure 1).

### 2.2. Data collection and follow-up

Trained physicians gathered clinical data from electronic medical records. The records contain information on the population, anthropometry, laboratory results, medical diagnoses, and procedures. After an overnight fast, venous blood samples were collected in the morning and examined the same day at the central laboratory using the standard procedures. After admission, all patients were routinely followed up by trained clinicians for major adverse cardiovascular and cerebrovascular events (MACCEs) and composite bleeding events at 3, 6, and 12 months and, then, at every 6 months; the longest individual follow-up period was 66 months. Follow-up data were obtained from hospital records or through telephone or in-person interviews with patients and their families.

The first observational endpoints of this study were MACCEs and composite bleeding events during the follow-up period of 47 (40, 54) months. We also analyzed the observational endpoints at the 24-month follow-up period after discharge (during the prolonged DAPT duration). MACCEs are defined as the composite of ischemic-driven revascularization, non-fatal ischemic stroke, non-fatal myocardial infarction (MI), cardiac death, and all-cause death. Bleeding Academic Research Composite bleeding events were created by combining BARC 3 or 5 types of bleeding events with BARC 1 or 2 types of bleeding events (17). Only the most serious event (all-cause death > non-fatal ischemic stroke > non-fatal MI > ischemia-driven revascularization) was chosen to perform our analysis for patients with multiple MACCEs occurring virtually and simultaneously throughout the follow-up. Similarly, BARC 3 or 5 types of bleeding events were selected for our analysis for patients who experienced BARC 3 or 5 types of bleeding events and BARC 1 or 2 types of bleeding events throughout the follow-up. Only the initial occurrence of the same event was intended to be used for our analysis of patients when it occurred more than once.

### 2.3. Definitions

According to the relevant guidelines, the diagnostic criteria for ACS included ST-segment elevation MI (STEMI) and non-ST-segment elevation ACS (NSTE-ACS) [non-ST-segment elevation MI (NSTEMI) or unstable angina (UA)] (6, 7). Patients were considered to have hypertension if they received treatment with a conclusive diagnosis or if their systolic blood pressure (SBP) was ≥140 mmHg or if their diastolic blood pressure (DBP) ≥90 mmHg was higher than two times on different days during the baseline hospitalization. According to the practical guidelines, patients with type 2 diabetes mellitus either had a prior, conclusive diagnosis or had the condition recently verified (18). Patients were considered to have hyperlipidemia if they received treatment with lipid-lowering medications or had fasting total cholesterol >6.22 mmol/L or low-density lipoprotein cholesterol (LDL-C) >4.14 mmol/L. Patients suffering from an ischemic stroke had a cerebral infarction or a transient ischemic attack. Patients with peripheral artery disease (PAD) had previously been diagnosed with artery disease as well as in the coronary and aortic arteries. They had 50% stenosis and/or signs of ischemia. Patients with the eGFR levels between 30 and 60 mL/min/1.73 m^2^ were considered to have renal dysfunction. Emergency PCI was defined as PCI performed within 24 h of hospital admission for patients with NSTE-ACS or within 12 h of symptom onset for patients with STEMI. Elective PCI was performed 24 h after hospital admission for—patients with NSTE-ACS or 12 h after symptom onset for patients with STEMI.

Weight (kg)/[height (m)]^2^ was the formula used to calculate body mass index (BMI). The formula for calculating the eGFR was 186 × serum creatinine (mg/dL)^−1.154^ × age^−0.203^ (× 0.742 if the patient was a woman) (19).

A number of the main coronary arteries, including the left anterior descending artery, the left circumflex artery, and the right coronary artery, must have a stenosis of ≥50% to be considered to have a several-vessel disease. Chronic total occlusion (CTO) lesions were defined as total obstruction persisting for more than 3 months, as determined by the coronary angiography findings or prior medical history. An individual stenotic lesion of over 20 mm is considered a diffuse lesion. The term “in-stent restenosis” (ISR) was used to describe a stenosis of ≥50% in a segment that was inside the stent or 5 mm away from it (20).

### 2.4. Statistical analysis

Continuous variables were presented as the mean and standard deviation or the median (IQR). A Mann–Whitney *U*-test or an independent-sample *t*-test was used to compare the two groups. Counts (percentages) were used to characterize categorical variables, which were then compared using either the Fisher's exact test or the Pearson chi-square test (Pearson χ^2^ test). Univariate and multivariable Cox proportional hazards analyses evaluated the predictive value of the variables for MACCEs and composite bleeding events. Several risk factors were present in the multivariate model, including clinically significant variables (*p* < 0.2) from the univariate model. The Kaplan–Meier survival curves estimated the cumulative incidence of MACCEs and composite bleeding events.

Further stratified analysis was performed to determine the prognostic impact of standard DAPT and prolonged DAPT for MACCEs and composite bleeding events. The propensity score for matching (PSM) was calculated using a binary logistic regression model, which took into account the use of statins, ACEI/ARB, β-blockers, P2Y_12_ inhibitors, and aspirin at the time of discharge. Finally, 986 standard patients with DAPT were individually matched at a ratio of 1:1 to patients with prolonged DAPT.

IBM SPSS Statistics (version 24.0) was used for data analysis. A statistically significant correlation was defined as a two-tailed *p*-value of <0.05.

## 3. Results

### 3.1. Basic characteristics of the standard and prolonged DAPT groups

The baseline characteristics of the standard and prolonged DAPT groups are displayed in Table 1. A total of 2,249 patients (60.98 ± 9.95 years; 23.5% women) were enrolled in the present study, with 1,011 (45.0%) in the standard DAPT group and 1,238 (55.0%) in the prolonged DAPT group. The baseline demographic characteristics, medical history, laboratory data, and angiographic information were similar between the two groups. Patients in the prolonged DAPT group had a higher proportion of β-blocker and angiotensin-converting enzyme inhibitor/angiotensin receptor blocker treatment at the time of discharge. There was no statistical difference in basic characteristics between the two groups after PSM (Supplementary Table 1).

**Table 1:** Baseline clinical characteristics of patients in the standard and prolonged DAPT groups.

**Characteristics**	**Total population**	**Standard DAPT group**	**Prolonged DAPT group**	***p*** **-value**
	**(*n* = 2,249)**	**(*n* = 1,124)**	**(*n* = 1,125)**	
Age, years	60.98 ± 9.95	60.83 ± 9.90	61.11 ± 9.98	0.502
Gender, men, *n* (%)	1,720 (76.5%)	768 (76.0%)	952 (76.9%)	0.603
BMI, kg/m^2^	23.28 ± 2.44	23.28 ± 2.46	23.28 ± 2.42	0.943
SBP, mmHg	128.28 ± 21.33	127.39 ± 21.04	129.00 ± 21.54	0.074
DBP, mmHg	77.66 ± 11.91	77.47 ± 11.74	77.81 ± 12.05	0.500
Heart rate, bpm	74.39 ± 10.03	73.94 ± 11.56	74.36 ± 12.40	0.103
Smoking history, *n* (%)	1,237 (55.0%)	560 (55.4%)	677 (54.7%)	0.738
Drinking history, *n* (%)	617 (27.4%)	295 (29.2%)	322 (26.0%)	0.094
Family history of CAD, *n* (%)	191 (8.5%)	89 (8.8%)	102 (8.2%)	0.633
Initial diagnosis, *n* (%)				0.218
UA	1,364 (60.6%)	633 (62.6%)	731 (59.0%)	
NSTEMI	163 (7.2%)	68 (6.7%)	95 (7.7%)	
STEMI	722 (32.1%)	310 (30.7%)	412 (33.3%)	
**Medical history**, ***n*** **(%)**				
Hypertension	1,297 (57.7%)	563 (55.7%)	734 (59.3%)	0.085
AF	61 (2.7%)	27 (2.7%)	34 (2.7%)	0.912
CHA2DS2-VASc score	2.00 (1.00, 3.00)	2.00 (1.00, 3.00)	2.00 (1.00, 3.00)	0.671
HAS-BLED score	1.00 (1.00, 2.50)	1.00 (1.00, 3.00)	1.00 (1.00, 2.25)	0.878
DM	599 (26.6%)	325 (32.1%)	359 (29.0%)	0.106
Dyslipidemia	251 (11.2%)	105 (10.4%)	146 (11.8%)	0.295
Renal dysfunction	45 (2.0%)	17 (1.7%)	28 (2.3%)	0.328
Previous MI	197 (8.8%)	79 (7.8%)	118 (9.5%)	0.152
Previous PCI	245 (10.9%)	112 (11.1%)	133 (10.8%)	0.799
Previous stroke	482 (21.4%)	200 (19.8%)	282 (22.8%)	0.085
Previous cerebral infarction	184 (8.2%)	80 (7.9%)	104 (8.4%)	0.675
Previous PAD	360 (16.0%)	156 (15.4%)	204 (16.5%)	0.500
**Laboratory results**				
WBC (× 109/L)	7.30 ± 2.55	7.33 ± 2.56	7.28 ± 2.54	0.580
PLT (× 109/L)	157.57 ± 55.69	155.30 ± 55.49	159.42 ± 55.80	0.081
Hb (g/L)	137.44 ± 18.49	137.82 ± 17.88	137.14 ± 18.97	0.386
BUN (mmol/L)	5.42 ± 1.95	5.36 ± 1.91	5.46 ± 1.98	0.259
Cr (umol/L)	68.50 ± 18.98	67.85 ± 18.28	68.63 ± 19.48	0.326
eGFR (mL/min/1.73 m^2^)	97.26 ± 28.63	98.47 ± 29.32	96.27 ± 28.03	0.070
FBG (mmol/L)	6.79 ± 2.92	6.86 ± 2.99	6.74 ± 2.86	0.338
HbA1c (%)	6.14 ± 1.28	6.20 ± 1.34	6.10 ± 1.22	0.068
HDL-C (mmol/L)	0.97 ± 0.22	0.98 ± 0.23	0.97 ± 0.22	0.108
TC (mmol/L)	3.72 ± 1.18	3.76 ± 1.18	3.69 ± 1.19	0.115
TG (mmol/L)	1.51 ± 0.88	1.50 ± 0.88	1.52 ± 0.88	0.588
LDL-C (mmol/L)	1.83 ± 0.86	1.87 ± 0.85	1.81 ± 0.87	0.089
NT-proBNP (pg/mL)	710.69 ± 1244.39	695.67 ± 1177.56	722.96 ± 1296.76	0.605
LVEF (%)	59.77 ± 11.35	59.84 ± 11.21	59.71 ± 11.46	0.779
**Angiographic data**				
LM disease, *n* (%)	264 (11.7%)	106 (10.5%)	158 (12.8%)	0.095
CTO, *n* (%)	661 (29.4%)	291 (28.8%)	370 (29.9%)	0.568
Number-vessel disease. *n* (%)				0.996
Single-vessel disease	561 (24.9%)	253 (25.0%)	308 (24.9%)	
Two-vessel disease	667 (29.7%)	300 (29.7%)	367 (29.6%)	
Three-vessel disease	1021 (45.4%)	458 (45.3%)	563 (45.5%)	
Diffuse lesion, *n* (%)	1390 (61.8%)	620 (61.3%)	770 (62.2%)	0.672
In-stent restenosis, *n* (%)	78 (3.5%)	35 (3.5%)	43 (3.5%)	0.988
Calcification lesion, *n* (%)	62 (2.8%)	30 (3.0%)	32 (2.6%)	0.582
Number of stents	1.76 ± 1.18	1.78 ± 1.19	1.74 ± 1.18	0.392
**Medication at the time of discharge**, ***n*** **(%)**				
ACEI/ARB	1,869 (83.1%)	812 (80.3%)	1,057 (85.4%)	0.001
β-blocker	1,839 (81.8%)	800 (79.1%)	1,039 (83.9%)	0.003
Statins	2,241 (99.6%)	1,006 (99.5%)	1,235 (99.8%)	0.480
P2Y12 inhibitor				0.120
Clopidogrel	1,954 (86.9%)	866 (85.7%)	1,088 (87.9%)	
Ticagrelor	295 (13.1%)	145 (14.3%)	150 (12.1%)	
Aspirin	2,249 (100.0%)	1,011 (100%)	1,238 (100%)	-
CRUSADE score	23.76 ± 10.90	23.39 ± 10.72	24.06 ± 11.05	0.147

DAPT, dual antiplatelet therapy; BMI, body mass index; SBP, systolic blood pressure; DBP, diastolic blood pressure; CAD, coronary artery disease; UA, unstable angina; NSTEMI, non ST-segment elevation myocardial infarction; STEMI, ST-segment elevation myocardial infarction; AF, atrial fibrillation; DM, diabetes mellitus; MI, myocardial infarction; PCI, percutaneous coronary intervention; PAD, peripheral artery disease; WBC, white blood cell; PLT, platelet; Hb, hemoglobin; BUN, blood urea nitrogen; Cr, creatinine; eGFR, estimated glomerular filtration rate; FBG, fasting blood glucose; HbA1c, glycosylated hemoglobin A1c; HDL-C, high-density lipoprotein-C; TC, total cholesterol; TG, triglyceride; LDL-C, low-density lipoprotein-C; LVEF, left ventricular ejection fraction; LM, left main; CTO, chronic total occlusion; ACEI, angiotensin-converting enzyme inhibitor; ARB, angiotensin receptor blocker.

### 3.2. Incidence of MACCEs in the standard and prolonged DAPT groups

A total of 271 (12.0%) MACCEs, including 112 (5.0%) all-cause deaths, 74 (3.3%) cardiac deaths, 14 (0.6%) non-fatal MIs, 33 (1.5%) non-fatal ischemic strokes, and 112 (5.0%) ischemia-driven revascularizations, were recorded at a median of 47 months of follow-up [47 (40, 54)]. No statistically significant difference in the prevalence of MACCEs (11.1% vs. 13.2% in those with standard DAPT, OR 0.828, 95% CI 0.642–1.068, *p* = 0.146), all-cause death (4.4 vs. 5.6% in those with standard DAPT, OR 0.778, 95% CI 0.532–1.138, *p* = 0.195), cardiac death (2.7 vs. 4.1% in those with standard DAPT, OR 0.648, 95% CI 0.407–1.033, *p* = 0.066), non-fatal MI (0.6 vs. 0.6% in those with standard DAPT, OR 1.089, 95% CI 0.377–3.150, *p* = 0.874), non-fatal ischemic stroke (1.4 vs. 1.6% in those with standard DAPT, OR 0.866, 95% CI 0.435–1.722, *p* = 0.681), and ischemia-driven revascularization (4.7 vs. 5.3% in those with standard DAPT, OR 0.871, 95% CI 0.596–1.274, *p* = 0.477) was observed between the two groups. A total of 133 (5.9%) MACCEs, including 70 (3.1%) all-cause deaths, 48 (2.1%) cardiac deaths, 6 (0.3%) non-fatal MIs, 11 (0.5%) non-fatal ischemic strokes, and 46 (2.0%) ischemia-driven revascularizations, were recorded at 24 months after discharge. No statistically significant difference in the prevalence of MACCEs (5.2 vs. 6.8% in those with standard DAPT, OR 0.744, 95% CI 0.524–1.057, *p* = 0.098), all-cause death (2.7 vs. 3.6% in those with standard DAPT, OR 0.764, 95% CI 0.475–1.232, *p* = 0.269), cardiac death (1.7 vs. 2.7% in those with standard DAPT, OR 0.629, 95% CI 0.353–1.119, *p* = 0.112), non-fatal MI (0.2 vs. 0.4% in those with standard DAPT, OR 0.407, 95% CI 0.074–2.227, *p* = 0.418), non-fatal ischemic stroke (0.4 vs. 0.6% in those with standard DAPT, OR 0.679, 95% CI 0.207–2.232, *p* = 0.736), and ischemia-driven revascularization (1.9 vs. 2.3% in those with standard DAPT, OR 0.813, 95% CI 0.454–1.458, *p* = 0.487) was found between the two groups (Table 2). There was no statistical difference in the incidence of MACCE and its components between the two groups after PSM (Supplementary Table 2).

**Table 2:** Incidence of MACCEs in the standard and prolonged DAPT groups.

**Endpoint event**	**Total population**	**Standard DAPT group**	**Prolonged DAPT group**	**OR (95%CI)**	***p*** **-value**
	**(*n* = 2,249)**	**(*n* = 1,011)**	**(*n* = 1,238)**		
**MACCE**, ***n*** **(%)**					
24 months	133 (5.9%)	69 (6.8%)	64 (5.2%)	0.744 (0.524, 1.057)	0.098
47 months^*^	271 (12.0%)	133 (13.2%)	138 (11.1%)	0.828 (0.642, 1.068)	0.146
**All-cause death**, ***n*** **(%)**					
24 months	70 (3.1%)	36 (3.6%)	34 (2.7%)	0.764 (0.475, 1.232)	0.269
47 months^*^	112 (5.0%)	57 (5.6%)	55 (4.4%)	0.778 (0.532, 1.138)	0.195
**Cardiac death**, ***n*** **(%)**					
24 months	48 (2.1%)	27 (2.7%)	21 (1.7%)	0.629 (0.353, 1.119)	0.112
47 months^*^	74 (3.3%)	41 (4.1%)	33 (2.7%)	0.648 (0.407, 1.033)	0.066
**Non-fatal MI**, ***n*** **(%)**					
24 months	6 (0.3%)	4 (0.4%)	2 (0.2%)	0.407 (0.074, 2.227)	0.418
47 months^*^	14 (0.6%)	6 (0.6%)	8 (0.6%)	1.089 (0.377, 3.150)	0.874
**Non-fatal ischemic stroke**, ***n*** **(%)**					
24 months	11 (0.5%)	6 (0.6%)	5 (0.4%)	0.679 (0.207, 2.232)	0.736
47 months^*^	33 (1.5%)	16 (1.6%)	17 (1.4%)	0.866 (0.435, 1.722)	0.681
**Ischemia-driven revascularization**, ***n*** **(%)**					
24 months	46 (2.0%)	23 (2.3%)	23 (1.9%)	0.813 (0.454, 1.458)	0.487
47 months^*^	112 (5.0%)	54 (5.3%)	58 (4.7%)	0.871 (0.596, 1.274)	0.477

* The median time of last follow-up was 47 months.

MACCE, major adverse cardiovascular and cerebrovascular events; DAPT, dual antiplatelet therapy; OR, odds ratio; CI, confidence interval; MI, myocardial infarction.

### 3.3. Cox proportional hazard analysis to assess the impact of MACCEs on prognosis

The relationship between DAPT duration and MACCEs was investigated using the Cox proportional hazard model. The DAPT duration was insignificantly related to MACCEs according to a univariate model (HR 0.848, 95% CI 0.668–1.076, *p* = 0.177) and a multivariate model (HR 0.813, 95% CI 0.638–1.036, *p* = 0.094) (Table 3). Meanwhile, the multivariate and univariate analyses revealed the absence of a meaningful relationship between the DAPT duration and cardiac death (HR 0.643, 95% CI 0.361–1.142, *p* = 0.132).

**Table 3:** Predictive value of DAPT duration for MACCE in Cox proportional hazard univariate and multivariate analyses.

**Characteristics**	**Univariate analysis**		**Multivariate analysis**	
	**HR (95%CI)**	***p*** **-value**	**HR (95%CI)**	***p*** **-value**
Age	1.025 (1.013, 1.038)	< 0.001	1.018 (1.003, 1.033)	0.017
Previous MI	1.563 (1.091, 2.240)	0.015		
Previous PCI	1.640 (1.184, 2.272)	0.003		
Previous stroke	1.030 (0.773, 1.372)	0.840	1.045 (0.777, 1.407)	0.771
AF	1.870 (1.071, 3.265)	0.028	1.656 (0.942, 2.909)	0.080
DM	1.246 (0.970, 1.600)	0.085		
Drinking history	0.809 (0.611, 1.071)	0.139		
DBP	0.987 (0.977, 0.997)	0.011		
HR	1.013 (1.004, 1.023)	0.007		
Number-vessel disease	1.329 (1.138, 1.554)	< 0.001		
LM disease	1.749 (1.284, 2.383)	< 0.001	1.469 (1.067, 2.022)	0.018
CTO	1.211 (0.940, 1.561)	0.139		
ISR	1.957 (1.181, 3.244)	0.009		
Number of stents	1.194 (1.089, 1.309)	< 0.001	1.114 (1.009, 1.229)	0.032
WBC	1.048 (1.003, 1.094)	0.036		
Hb	0.991 (0.984, 0.997)	0.003	0.993 (0.986, 1.000)	0.047
LVEF	0.977 (0.967, 0.986)	< 0.001	0.987 (0.976, 0.998)	0.019
BUN	1.094 (1.035, 1.156)	0.001		
Cr	1.007 (1.002, 1.013)	0.005		
eGFR	0.997 (0.993, 1.001)	0.167		
FBG	1.045 (1.007, 1.083)	0.019		
HbA1c	1.187 (1.099, 1.282)	< 0.001	1.200 (1.068, 1.348)	0.002
HDL-C	0.618 (0.356, 1.073)	0.087		
LDL-C	1.125 (0.992, 1.276)	0.066	1.161 (1.018, 1.324)	0.026
DAPT duration	0.848 (0.668, 1.076)	0.177	0.813 (0.638, 1.036)	0.094
ACEI/ARB	0.768 (0.543, 1.087)	0.136		
β-blocker	0.584 (0.404, 0.844)	0.003		

DAPT, dual antiplatelet therapy; MACCE, major adverse cardiovascular and cerebrovascular events; HR, hazard ratio; CI, confidence interval; MI, myocardial infarction; PCI, percutaneous coronary intervention; AF, atrial fibrillation; DM, diabetes mellitus; DBP, diastolic blood pressure; HR, heart rate; LM, left main; CTO, chronic total occlusion; ISR, in-stent restenosis; WBC, white blood cell; Hb, hemoglobin; LVEF, left ventricular ejection fraction; BUN, blood urea nitrogen; Cr, creatinine; eGFR, estimated glomerular filtration rate; FBG, fasting blood glucose; HbA1c, glycosylated hemoglobin A1c; HDL-C, high-density lipoprotein cholesterol; LDL-C, low-density lipoprotein cholesterol; ACEI, angiotensin-converting enzyme inhibitor; ARB, angiotensin receptor blocker.

### 3.4. Sensitivity analysis

**Figure 2:**
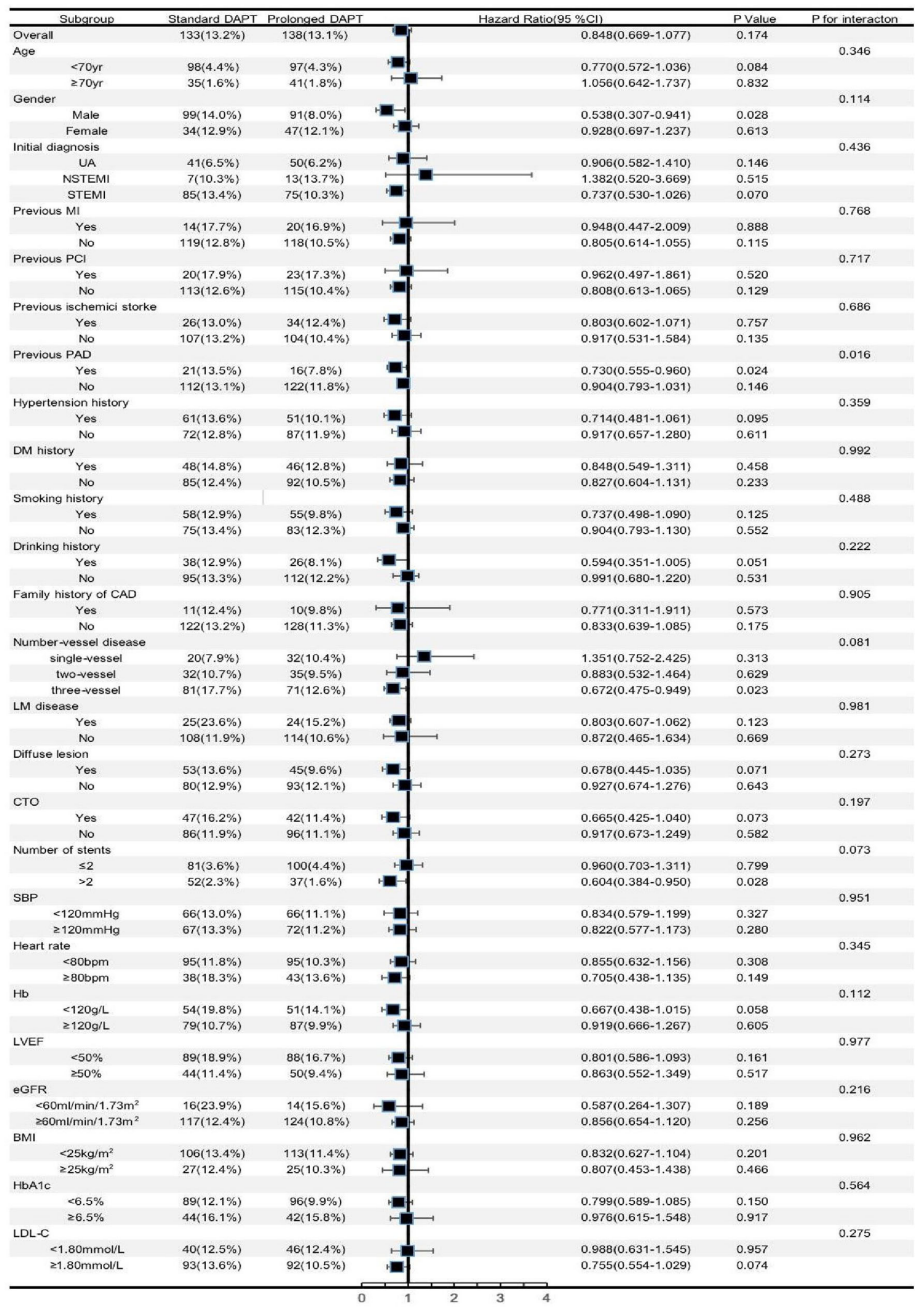
Forest plot investigating the association between the DAPT duration and MACCEs in different subgroups. DAPT, dual antiplatelet therapy; UA, unstable angina; NSTEMI, non ST-segment elevation myocardial infarction; STEMI, ST-segment elevation myocardial infarction; MI, myocardial infarction; PCI, percutaneous coronary intervention; PAD, peripheral artery disease; DM, diabetes mellitus; CAD, coronary artery disease; LM, left main; CTO, chronic total occlusion; SBP, systolic blood pressure; Hb, hemoglobin; LVEF, left ventricular ejection fraction; eGFR, estimated glomerular filtration rate; BMI, body mass index; HbA1c, glycosylated hemoglobin A1c; LDL-C, low-density lipoprotein cholesterol; CJ, confidence interval.

We further analyzed different subgroups to evaluate the independent association of DAPT duration with MACCEs. According to Figure 2, being men (HR 0.538, 95% CI 0.307–0.941, *p* = 0.028), having a history of PAD (HR 0.730, 95% CI 0.555–0.960, *p* = 0.024), having three-vessel disease (HR 0.672, 95% CI 0.475–0.949, *p* = 0.023), and having >2 stents implanted (HR 0.604, 95% CI 0.384–0.950, *p* = 0.028) primarily reflected the significant predictive effect of DAPT duration on MACCEs. It is worth noting that patients with a history of PAD appeared to have a higher predictive value for DAPT duration [HR (95%CI) with previous PAD 0.730 (0.555–0.960) vs. without previous PAD 0.904 (0.793–1.031), *p* for interaction = 0.016].

### 3.5. Incidence of composite bleeding events in the standard and prolonged DAPT groups

A total of 243 (10.8%) composite bleeding events, including 46 (2.0%) BARC 3 or 5 types of bleeding events and 197 (8.8%) BARC 1 or 2 types of bleeding events, were recorded at a median of 47 months of follow-up [47 (40, 54)]. The incidence of composite bleeding events (13.2 vs. 7.9% in those with standard DAPT, OR 1.765, 95% CI 1.332–2.338, *p* < 0.001), BARC 3 or 5 types of bleeding events (3.0 vs. 0.9% in those with standard DAPT, OR 3.430, 95% CI 1.648–7.141, *p* < 0.001), and BARC 1 or 2 types of bleeding events (10.2 vs. 7.0% in those with standard DAPT, OR 1.500, 95% CI 1.107–2.032, *p* = 0.008) increased significantly in the prolonged DAPT group. A total of 143 (6.4%) composite bleeding events, including 24 (1.1%) BARC 3 or 5 types of bleeding events and 119 (5.3%) BARC 1 or 2 types of bleeding events, were recorded at 24 months after discharge, and the incidence of composite bleeding events (9.3 vs. 2.8% in those with standard DAPT, OR 3.597, 95% CI 2.358–5.495, *p* < 0.001), BARC 3 or 5 types of bleeding events (1.3 vs. 0.6% in those with standard DAPT, OR 2.469, 95% CI 1.001–6.250, *p* = 0.048), and BARC 1 or 2 types of bleeding events (7.8 vs. 2.2% in those with standard DAPT, OR 3.817, 95% CI 2.387–6.135, *p* < 0.001) also increased significantly in the prolonged DAPT group (Table 4). The incidence of composite bleeding events and its components was statistically different between the two groups after PSM (Supplementary Table 3).

**Table 4:** Incidence of composite bleeding events in the standard and prolonged DAPT groups.

**Endpoint event**	**Total population** **(*n* = 2,249)**	**Standard DAPT group** **(*n* = 1,011)**	**Prolonged DAPT group** **(*n* = 1,238)**	**OR (95%CI)**	***p*** **-value**
**Composite bleeding events**, ***n*** **(%)**					
24 months	143 (6.4%)	28 (2.8%)	115 (9.3%)	3.597 (2.358, 5.495)	<0.001
47 months^*^	243 (10.8%)	80 (7.9%)	163 (13.2%)	1.765 (1.332, 2.338)	<0.001
**BARC 3 or 5 bleeding events**, ***n*** **(%)**					
24 months	24 (1.1%)	6 (0.6%)	18 (1.3%)	2.469 (1.001, 6.250)	0.048
47 months^*^	46 (2.0%)	9 (0.9%)	37 (3.0%)	3.430 (1.648, 7.141)	<0.001
**BARC 1 or 2 bleeding events**, ***n*** **(%)**					
24 months	119 (5.3%)	22 (2.2%)	97 (7.8%)	3.817 (2.387, 6.135)	<0.001
47 months^*^	197 (8.8%)	71 (7.0%)	126 (10.2%)	1.500 (1.107, 2.032)	0.008

* The median time of the last follow-up was 47 months.

DAPT, dual antiplatelet therapy; OR, odds ratio; CI, confidence interval; BARC, bleeding academic research consortium.

### 3.6. Cox proportional hazard analysis to assess the impact of composite bleeding events on prognosis

The relationship between DAPT duration and composite bleeding events was investigated using the Cox proportional hazard model. The DAPT duration was substantially related to composite bleeding events according to a univariate model (HR 1.724, 95% CI 1.319–2.252, *p* < 0.001). The other significant risk factors included sex, WBC count, Hb level, LVEF, HbA1c level, previous history of MI, and use of ticagrelor. The multivariate model for analysis included various risk factors, such as significant variables (*p*<0.2) from the univariate model, and the DAPT duration remained an independent predictor of composite bleeding events (HR 1.704, 95% CI 1.302–2.232, *p* < 0.001). The other independent predictors included age, Hb, and HbA1c, a previous history of diabetes mellitus (DM), and the use of ticagrelor (Table 5). Meanwhile, the multivariate and univariate analyses indicated that a meaningful relationship between DAPT duration and BARC 3 or 5-type bleeding events exists (Supplementary Table 4).

**Table 5:** Predictive value of DAPT duration for composite bleeding events in Cox proportional hazard univariate and multivariate analysis.

**Characteristics**	**Univariate analysis**		**Multivariate analysis**	
	**HR (95%CI)**	***p*** **-value**	**HR (95%CI)**	***p*** **-value**
Gender	1.447 (1.101, 1.902)	0.008		
Age	1.008 (0.996, 1.020)	0.107	1.017 (1.003, 1.032)	0.019
Previous MI	2.032 (1.110, 3.717)	0.022		
Hypertension	1.207 (0.931, 1.564)	0.155		
DM	1.260 (0.968, 1.639)	0.085	1.749 (1.256, 2.437)	0.001
Smoking	1.245 (0.969, 1.603)	0.087		
DBP	0.993 (0.982, 1.003)	0.169		
SBP	0.995 (0.990, 1.001)	0.124		
WBC	1.063 (1.007, 1.122)	0.028		
PLT	0.998 (0.995, 1.001)	0.122		
Hb	0.987 (0.981, 0.994)	<0.001	0.987 (0.979, 0.995)	0.002
LVEF	0.986 (0.975, 0.998)	0.020		
FBG	1.049 (0.999, 1.101)	0.055		
HbA1c	1.125 (1.004, 1.259)	0.042	1.229 (1.043, 1.447)	0.014
P2Y12 inhibitor	1.808 (1.325, 2.468)	<0.001	1.843 (1.342, 2.533)	<0.001
DAPT duration	1.724 (1.319, 2.252)	<0.001	1.704 (1.302, 2.232)	<0.001

DAPT, dual antiplatelet therapy; HR, hazard ratio; CI, confidence interval; MI, myocardial infarction; DM, diabetes mellitus; DBP, diastolic blood pressure; SBP, systolic blood pressure; WBC, white blood cell; PLT, platelet; Hb, hemoglobin; LVEF, left ventricular ejection fraction; FBG, fasting blood glucose; HbA1c, glycosylated hemoglobin A1c.

### 3.7. Sensitivity analysis

**Figure 3:**
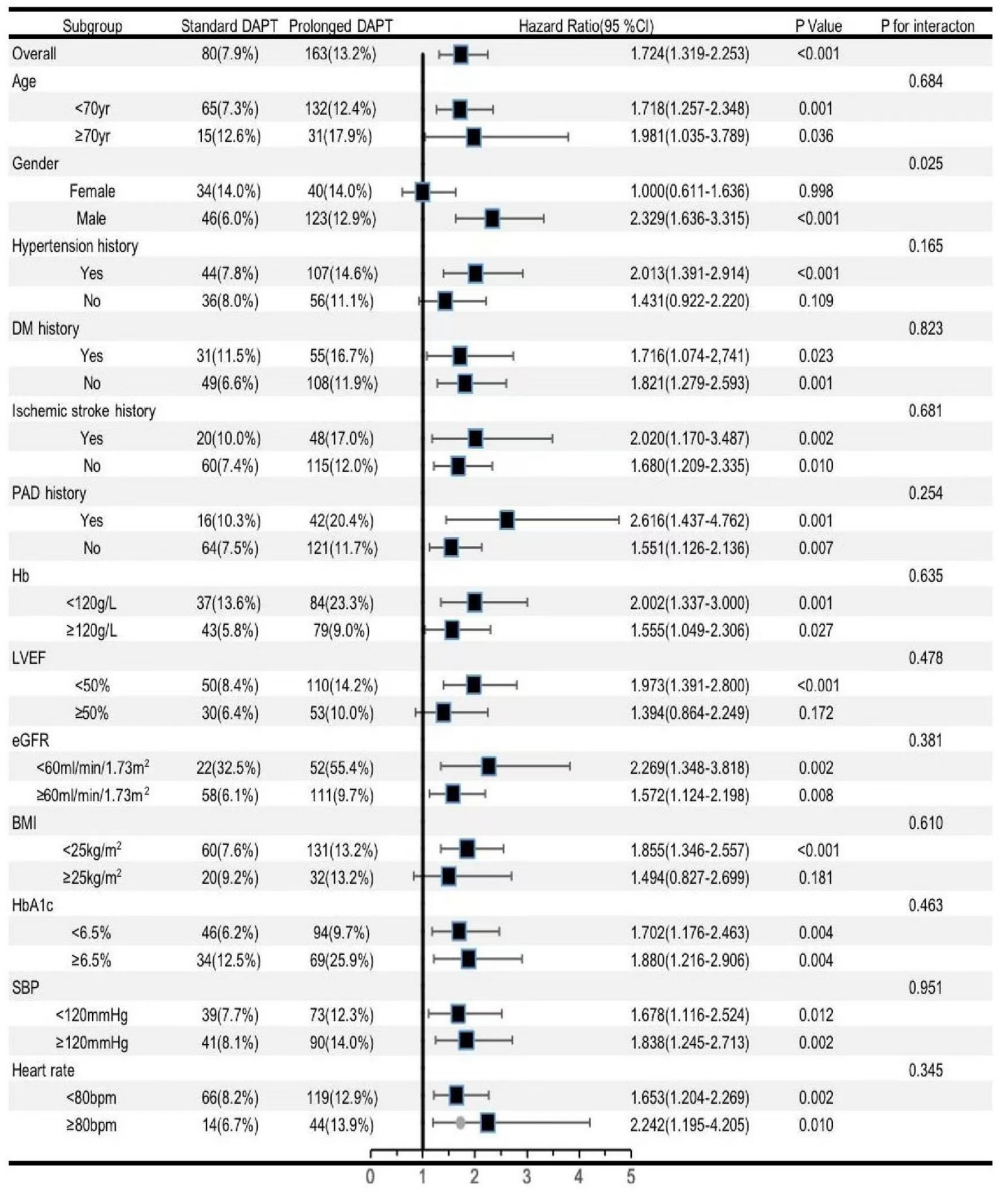
Forest plot investigating th e association between the DA PT duration and composite bleeding events in different subgroups. DAPT, dual antiplatelet therapy; DM, diabetes mellitus; PAD, peripheral artery disease; Hb, hemoglobin; LVEF, left ventricular ejection fraction; eGFR, estimated glomerular filtration rate; BMI, body mass index; HbA1c, glycosylated hemoglobin A1c; SEP, systolic blood pressure; Cl, confidence interval.

We further analyzed different subgroups to evaluate the independent association of DAPT duration with composite bleeding events. According to Figure 3, the significant predictive effect of DAPT duration on composite bleeding events was primarily reflected in the subgroups of patients aged < 70 years and patients aged ≥70 years, patients who were men, patients with a history of hypertension, patients with and without a history of DM, patients who experienced ischemic stroke, and patients with a history of PAD, Hb ≥120 g/L and <120 g/L, LVEF <50%, eGFR <60 ml/min/1.73 m^2^ and ≥60 ml/min/1.73 m^2^, BMI <25 kg/m^2^, HbA1c <6.5% and ≥6.5%, SBP ≥120 mmHg and <120 mmHg, and heart rate ≥80 bpm and <80 bpm. Female patients appeared to have a higher predictive value for DAPT duration [HR (95%CI) for women vs. men = 2.329 (1.636–3.315) vs. 1.000 (0.611–1.636), *p* for interaction =0.025].

## 4. Discussion

In this cohort of Chinese patients with ACS who were treated with emergency PCI with drug-eluting stents, the prolonged DAPT group had a significantly higher risk of composite bleeding events than the standard DAPT group. DAPT duration was an independent predictor of composite bleeding events. However, we did not find a statistically significant difference in the prevalence of MACCEs between the two groups, and DAPT duration was not an independent predictor of MACCEs. To the best of our knowledge, this study is the first to examine the benefits and risks of prolonged DAPT in Chinese patients with ACS who underwent emergency PCI with DES.

STEMI, NSTEMI, and UA are clinical diagnoses caused by acute myocardial ischemia, which is referred to as ACS. Several studies confirmed that the pathophysiology of ACS includes coronary vulnerable plaque rupture, vasospasm, and vascular endothelial dysfunction caused by oxidative damage and inflammation, which result in platelet activation, adhesion, aggregation, and secondary thrombosis (21). Patients who have experienced an ACS are at a higher risk of having recurrent ischemic events. The EPICOR Asia study enrolled 12,922 patients with ACS [mostly from China (63.6%)]: 6,616 (51.2%) patients with STEMI, 2,570 (19.9%) patients with NSTEMI, and 3,736 (28.9%) patients with UA. The study showed that all-cause mortality during the 2-year follow-up period was 5.2%, and the composite endpoint of death, MI, and stroke during the 2-year follow-up period was 8.4% (22). In the present study, all-cause mortality during the 24-month follow-up period was 3.1%, and the composite endpoint of death, non-fatal MI, non-fatal ischemic stroke, and ischemia-driven revascularization during the 24-month follow-up period was 5.9%. The lower risk of an adverse event in the present study may be due to the higher proportion of UA. Therefore, enhanced antiplatelet therapy is important for preventing and treating thrombosis. In recent years, new P2Y_12_ inhibitors (prasugrel and ticagrelor) have been affirmed by many large-scale clinical studies and have been recommended by guidelines due to their more powerful antiplatelet effect. However, new P2Y_12_ inhibitors (prasugrel and ticagrelor) may increase the incidence of bleeding events in East Asians as opposed to clopidogrel, according to various studies. A Korean study enrolled 4,421 patients (637 patients prescribed prasugrel and 3,784 patients prescribed clopidogrel) with acute MI who underwent successful revascularization. No statistically significant difference was detected between prasugrel and clopidogrel in the composite ischemic events of cardiac death, MI, stroke, or target vessel revascularization at 6 months (2.4 vs. 2.9%, *p* = 0.593). Compared with clopidogrel, prasugrel increased the presence of nosocomial thrombolysis in myocardial infarction (TIMI) major or minor bleeding (5.3 vs. 2.7%, *p* = 0.015) (23). Meanwhile, a Korean study enrolled 800 patients with ACS accepted for PCI management. No statistically significant difference between ticagrelor and clopidogrel was found in the composite ischemic events of cardiac death, MI, or stroke at 12 months (9.2 vs. 5.8%; HR, 1.62; *p* = 0.593). Compared with clopidogrel, ticagrelor increased the prevalence rates of clinically significant bleeding (11.7 vs. 5.3%; HR, 2.26; *p* = 0.002), major bleeding (7.5 vs. 4.1%, *p* = 0.04), and fatal bleeding (1.0 vs. 0.0%, *p* = 0.04) (24). As in the current research, the proportion of clopidogrel treatment was up to 86.9%, and the proportion of ticagrelor was only 13.1% during the DAPT period. Furthermore, the proportion of ticagrelor in the two groups was similar (14.3 vs. 12.1%, *p* = 0.120). The multivariate analysis based on the Cox proportional hazard model showed that the use of ticagrelor was insignificantly associated with MACCEs, cardiac death, or BARC 3 or 5 bleeding events. However, the use of ticagrelor was an independent predictor of composite bleeding events.

Recent guidelines in Europe and the United States advocate DAPT combined with aspirin and a P2Y_12_ inhibitor (clopidogrel, prasugrel, and ticagrelor) for up to 12 months after ACS to lower the risk of ischemic events, such as recurrent MI and ST (6–8). However, the risk of target lesion failure is still 2–4% annually after 1 year of DAPT (25). The guidelines in Europe recommend DAPT at >12 months in patients at a high risk of ischemic events and without an increased risk of major bleeding (Class IIa indication) (6). The EPICOR Asia trial showed that 78.8% of patients with NSTEMI continued DAPT for over 12 months (26). In the present study, 55.0% of patients with ACS continued DAPT at 12–24 months. A study enrolled 9,961 patients (5,020 patients accepted prolonged DAPT and 4,941 patients accepted standard DAPT) after they underwent successful revascularization with DES, and the study showed that the incidence of MACCEs (4.3 vs. 5.9%; OR, 0.71; *p* < 0.001), MI (2.1 vs. 4.1%; OR, 0.47; *p* < 0.001), and ST (0.4 vs. 1.4%; OR, 0.29; *p* < 0.001) was significantly lower in the prolonged DAPT group compared with the standard group. However, the incidence of severe or moderate bleeding events was elevated with prolonged DAPT (2.5 vs. 1.6%; *p* = 0.001) (10). The PEGASUS-TIMI 54 trial demonstrated that TIMI severe bleeding events were much more prevalent in the prolonged group (2.5 vs. 1.1%; OR, 2.36; *p* < 0.001); however, the composite ischemic events of cardiovascular death, MI, or stroke were less common in the prolonged DAPT group (7.9 vs. 9.6%; OR, 0.80; *p* = 0.001) (27). EPICOR Asia showed that the composite endpoint occurred less frequently in the prolonged DAPT group (3.1 vs. 10.6%), and only four patients had severe bleeding events in the prolonged DAPT group (26). ARCTIC interruption showed that the incidence of endpoints had no statistically significant difference between the standard DAPT and prolonged DAPT groups (4.0 vs. 4.0%; HR, 1.17; *p* = 0.58), and either minor or severe bleeding was much more common in the prolonged DAPT group than in the standard DAPT group (2.0 vs. 1.0%; OR, 0.26; *p* = 0.04) (12). To evaluate the benefit and risk of prolonged DAPT for predicting MACCEs and composite bleeding events in patients with ACS who underwent emergency PCI with DES, we analyzed a cohort of 2,249 Chinese patients with ACS and found no statistically significant difference between the standard DAPT and prolonged DAPT groups regarding the incidence of MACCEs. However, the prolonged DAPT group experienced significantly more composite bleeding events than the standard DAPT group, and DAPT duration was an independent predictor of composite bleeding events. Compared with the EPICOR Asia study, prolonged DAPT duration did not reduce the incidence of MACCEs in the present study due to enrolling a higher proportion of UA.

The risk of ischemia and bleeding needs to be evaluated when prolonging DAPT. A previous study showed that the risk factors for ischemic events included older age, ACS, previous MI, complex coronary artery disease (≥3 stents implanted, ≥3 lesions treated, LM, bifurcation, CTO, and previous ST on antiplatelet treatment), DM, PAD, and chronic kidney disease (CKD) (3, 7). The risk factors for bleeding events included a previous history of intracerebral hemorrhage or gastrointestinal bleeding, a previous history of moderate or severe ischemic stroke, a history of consuming oral anticoagulants, being women, being of older age, patients with low weight, patients with CKD, patients with liver failure, patients with anemia, and patients with long-term treatment with steroids or non-steroidal anti-inflammatory drugs (NSAIDs) (3, 7). As part of the current research, we analyzed a cohort of 2,249 Chinese patients with ACS. The independent predictors of MACCEs included age, Hb, LVEF, HbA1c, LDL-C, LM disease, and the number of stents implanted. Meanwhile, the independent predictors of composite bleeding events included age, DM, Hb, HbA1c, and use of ticagrelor. In the subgroup analysis, we found that prolonged DAPT could significantly reduce the risk of MACCEs in the three-vessel disease and being men compared with standard DAPT. Meanwhile, we discovered that prolonged DAPT had no effect on the risk of composite bleeding events in women without a prior history of hypertension, patients with LVEF ≥50%, and patients with BMI ≥25 kg/m^2^ compared with standard DAPT.

This study has several limitations that should be acknowledged. First, this study is an observational, retrospective, single-center study. Therefore, this trial could not determine the benefits or risks of prolonged DAPT in Chinese patients with ACS after emergency PCI with DES. Second, the sample size and the follow-up period might be insufficient. Third, our study's exclusion of patients with ACS who underwent elective PCI or were treated with a drug-coated balloon (DCB) may have limited the generalizability of our findings to patients with ACS who underwent primary PCI with DES and may have resulted in selection bias.

## 5. Conclusion

Compared with the standard DAPT group, the prolonged DAPT group had a statistically significant higher prevalence of composite bleeding events. However, the incidence of MACCEs showed no statistically significant difference between the two groups. In the subgroup analysis, prolonged DAPT significantly reduced the risk of MACCEs in the three-vessel disease and male subgroups. In contrast, prolonged DAPT did not increase the risk of composite bleeding events in men with no prior history of hypertension or DM, LVEF ≥50%, and BMI ≥25 kg/m^2^ subgroups. Additional large-scale, prospective cohort, multicenter studies with a sizable sample and a prolonged follow-up period will be needed to support our findings.

## Data availability statement

The original contributions presented in the study are included in the article/Supplementary material, further inquiries can be directed to the corresponding author.

## Ethics statement

The studies involving human participants were reviewed and approved by the Academic Committee of the First Affiliated Hospital of Xi'an Jiaotong University. The patients/participants provided their written informed consent to participate in this study.

## Author contributions

YZ and NG designed and drafted the manuscript. Y-bL, YZ, and ZZ carried out the cohort's follow-up and gathered the data. CC and F-FN evaluated the data and edited the text. YZ, ZZ, and F-FN did the planning and coordination of the research. The final manuscript was reviewed and approved by all authors.
