# Supplementary material for: Prolonged dual antiplatelet therapy for Chinese ACS patients undergoing emergency PCI with drug-eluting stents: Benefits and risks
Source: Front Cardiovasc Med. 2023 Feb 9;10:1080673. doi: 10.3389/fcvm.2023.1080673 (PMC9976624; doi:10.3389/fcvm.2023.1080673)
Supplement: Supplementary file 1 [file Table_1.docx]

**Supplemental Table 1 Baseline clinical characteristics of patients in the standard and prolonged DAPT groups after propensity score matching**

| Characteristics | Standard DAPT group | Prolonged DAPT group | p value |
| --- | --- | --- | --- |
|  | (n=986) | (n=986) |  |
| Age, years | 60.81±9.87 | 61.09±9.91 | 0.525 |
| Gender, male, n (%) | 752（76.3%） | 769（78.0%） | 0.362 |
| BMI, kg/m2 | 23.29±2.47 | 23.28±2.39 | 0.954 |
| SBP, mmHg | 127.46±21.12 | 129.32±21.80 | 0.055 |
| DBP, mmHg | 77.50±11.71 | 78.02±12.24 | 0.335 |
| Heart rate, bpm | 73.96±11.59 | 74.83±12.57 | 0.400 |
| Smoking history, n (%) | 547（55.5%） | 543（55.1%） | 0.856 |
| Drinking history, n (%) | 289（29.3%） | 261（26.5%） | 0.160 |
| Family history of CAD, n (%) | 86（8.7%） | 93（9.4%） | 0.583 |
| Initial diagnosis, n (%) |  |  | 0.354 |
| UA | 617（62.6%） | 587（59.5%） |  |
| NSTEMI | 68（6.9%) | 78（7.9%） |  |
| STEMI | 301（30.5%） | 321（32.6%） |  |
| Medical history, n (%) |  |  |  |
| Hypertension | 551（55.9%） | 587（59.5%） | 0.101 |
| AF | 25 (2.5%) | 31 (3.1%) | 0.416 |
| CHA_2_DS_2_-VASc score | 2.00（1.00，3.00） | 2.00（1.00，3.00） | 0.600 |
| HAS-BLED score | 1.00（1.00，3.00） | 1.00（1.00，2.50） | 0.780 |
| DM | 316（32.0%） | 296（30.0%） | 0.330 |
| Dyslipidemia | 102（10.4%） | 116（11.8%） | 0.319 |
| Renal dysfunction | 16（1.6%） | 27（2.7%） | 0.090 |
| Previous MI | 78（7.9%） | 95（9.6%） | 0.176 |
| Previous PCI | 107（10.9%） | 104（10.5%） | 0.821 |
| Previous stroke | 195（19.8%） | 227（23.0%） | 0.079 |
| Cerebral infarction | 78 (7.9%) | 82 (8.3%) | 0.749 |
| Previous PAD | 152（15.4%） | 167（16.9%） | 0.359 |
| Laboratory results |  |  |  |
| WBC ( ×109/L) | 7.32 ± 2.52 | 7.29 ± 2.53 | 0.814 |
| PLT ( ×109/L) | 154.96 ± 55.19 | 158.76 ± 54.63 | 0.125 |
| Hb (g/L) | 137.88 ± 17.81 | 136.93 ± 19.00 | 0.249 |
| BUN (mmol/L) | 5.36 ± 1.92 | 5.42 ± 1.95 | 0.483 |
| Cr (umol/L) | 68.35 ± 19.76 | 69.18 ± 18.48 | 0.339 |
| eGFR (mL/min/1.73m2) | 98.14 ± 29.30 | 98.01 ± 27.29 | 0.914 |
| FBG (mmol/L) | 6.87 ± 3.01 | 6.72 ± 2.89 | 0.247 |
| HbA1C (%) | 6.21 ± 1.35 | 6.10 ± 1.20 | 0.052 |
| HDL-C (mmol/L) | 0.98 ± 0.23 | 0.96 ± 0.22 | 0.070 |
| TC (mmol/L) | 3.77 ± 1.18 | 3.68 ± 1.21 | 0.054 |
| TG (mmol/L) | 1.50 ± 0.87 | 1.50 ± 0.84 | 0.931 |
| LDL-C (mmol/L) | 1.87 ± 0.86 | 1.80 ± 0.88 | 0.055 |
| NT-proBNP (pg/mL) | 695.01 ± 1174.15 | 732.08 ± 1311.63 | 0.509 |
| LVEF (%) | 59.80 ± 11.22 | 59.67 ± 11.46 | 0.807 |
| Angiographic data |  |  |  |
| LM disease, n (%) | 102（10.3%） | 122（12.0%） | 0.172 |
| CTO, n (%) | 284（28.8%） | 195（29.9%） | 0.586 |
| Number-vessel disease. n (%) |  |  | 0.895 |
| Single-vessel disease | 249（25.3%） | 240（24.3%） |  |
| Two-vessel disease | 291（29.5%） | 295（29.9%） |  |
| Three-vessel disease | 446（45.2%） | 451（45.7%） |  |
| Diffuse lesion, n (%) | 604（61.3%） | 610（61.9%） | 0.781 |
| In-stent restenosis, n (%) | 33（3.3%） | 34（3.4%） | 0.901 |
| Calcification lesion, n (%) | 29（2.9%） | 26（2.6%） | 0.682 |
| Number of stents | 1.79 ± 1.19 | 1.75 ± 1.18 | 0.541 |
| Medication at discharge, n (%) |  |  |  |
| ACEI/ARB | 807（81.8%） | 813（82.5%） | 0.724 |
| β-blocker | 781（79.2%） | 793（80.4%） | 0.501 |
| Statins | 985（99.9%） | 985（99.9%） | >0.999 |
| P2Y_12_ inhibitor |  |  | \| 0.330 \| \| --- \| |
| Clopidogrel | 856（86.8%） | 841（85.3%） |  |
| Ticagrelor | 130（13.2%） | 145（14.7%） |  |
| Aspirin | 986（100%） | 986（100%） | - |
| CRUSADE score | 23.48 ± 10.75 | 23.54 ± 10.86 | 0.902 |

*DAPT: dual antiplatelet therapy; BMI: body mass index; SBP: systolic blood pressure; DBP: diastolic blood pressure; CAD: coronary artery disease; UA: unstable angina; NSTEMI: non ST-segment elevation myocardial infarction; STEMI: ST-segment elevation myocardial infarction; AF: atrial fibrillation; DM: diabetes mellitus; MI: myocardial infarction; PCI: percutaneous coronary intervention; PAD: peripheral artery disease; WBC: white blood cell; PLT: platelet; Hb: hemoglobin; BUN: blood urea nitrogen; Cr: creatinine; eGFR, estimated glomerular filtration rate; FBG: fasting blood gulcose; HbA1C: glycosylated hemoglobin A1c; HDL-C: high-density lipoprotein-C; TC: total cholesterol; TG: triglyceride; LDL-C: low-density lipoprotein-C; LVEF: left ventricular ejection fracion; LM: left main; CTO: chronic total occlusion; ACEI: angiotensin converting enzyme inhibitor; ARB: angiotensin receptor blocker.*
