# Supplementary material for: Prolonged dual antiplatelet therapy for Chinese ACS patients undergoing emergency PCI with drug-eluting stents: Benefits and risks
Source: Front Cardiovasc Med. 2023 Feb 9;10:1080673. doi: 10.3389/fcvm.2023.1080673 (PMC9976624; doi:10.3389/fcvm.2023.1080673)
Supplement: Supplementary file 2 [file Table_2.docx]

**Supplemental Table 2 Incidence of MACCEs in the standard and prolonged DAPT groups after propensity score matching**

| Endpoint event | Total population | Standard DAPT group | Prolonged DAPT group | OR（95%CI） | p value |
| --- | --- | --- | --- | --- | --- |
|  | (n=1972) | (n=986) | (n=986) |  |  |
| MACCE, n (%) |  |  |  |  |  |
| 24months | 119（6.0%) | 67（6.8%) | 52（5.3%) | 0.764（0.526，1.110） | 0.156 |
| 47months* | 237（12.0%) | 129（13.1%) | 108（11.0%) | 0.817（0.622，1.073） | 0.146 |
| All-cause death, n (%) |  |  |  |  |  |
| 24months | 64（3.2%) | 34（3.4%) | 30（3.0%) | 0.879（0.533，1.447） | 0.611 |
| 47months* | 101（5.1%) | 54（5.5%) | 47（4.8%) | 0.864（0.578，1.291） | 0.475 |
| Cardiac death, n (%) |  |  |  |  |  |
| 24months | 44（2.2%) | 26（2.6%) | 18（1.8%) | 0.689（0.374，1.261） | 0.223 |
| 47months* | 65（3.3%) | 39（4.0%) | 26（2.6%) | 0.658（0.397，1.089） | 0.101 |
| Nonfatal MI, n (%) |  |  |  |  |  |
| 24months | 5（0.3%) | 4（0.4%) | 1（0.1%) | 0.249（0.028，2.232） | 0.374 |
| 47months* | 11（0.6%) | 6（0.6%) | 5（0.5%) | 0.832（0.253，2.737） | 0.762 |
| Nonfatal ischemic stroke, n (%) |  |  |  |  |  |
| 24months | 9（0.5%) | 6（0.6%) | 3（0.3%) | 0.499（0.124，2.000） | 0.316 |
| 47months* | 27（1.4%) | 16（1.6%) | 11（1.1%) | 0.684（0.316，1.481） | 0.333 |
| Ischemia-driven revacularzation, n (%) |  |  |  |  |  |
| 24months | 41（2.1%) | 23（2.3%) | 18（1.8%) | 0.779（0.418，1.451） | 0.430 |
| 47months* | 98（5.0%) | 53（5.4%) | 45（4.6%) | 0.842（0.560，1.265） | 0.407 |

***：the median time of last follow-up was 47 months

*MACCE: major adverse cardiovascular and cerebrovascular events; DAPT: dual antiplatelet therapy; OR: odds ratio; CI: confidence interval; MI: myocardial infarction.*
