# Supplementary material for: Prolonged dual antiplatelet therapy for Chinese ACS patients undergoing emergency PCI with drug-eluting stents: Benefits and risks
Source: Front Cardiovasc Med. 2023 Feb 9;10:1080673. doi: 10.3389/fcvm.2023.1080673 (PMC9976624; doi:10.3389/fcvm.2023.1080673)
Supplement: Supplementary file 3 [file Table_3.docx]

**Supplemental Table 3 Incidence of composite bleeding events in the standard and prolonged DAPT groups after propensity score matching**

| Endpoint event | Total population | Standard DAPT group | Prolonged DAPT group | OR（95%CI） | p value |
| --- | --- | --- | --- | --- | --- |
|  | (n=1976) | (n=986) | (n=986) |  |  |
| Composite bleeding events, n (%) |  |  |  |  |  |
| 24months | 123（6.2%) | 26（2.6%) | 97（9.8%) | 4.032（2.591，6.289） | <0.001 |
| 47months* | 211（10.7%) | 73（7.4%) | 138（14.0%) | 2.035（1.510，2.744） | <0.001 |
| BARC 3 or 5 bleeding events, n (%) |  |  |  |  |  |
| 24months | 20（1.0%) | 5（0.5%) | 15（1.5%) | 3.030（1.098，8.403） | 0.025 |
| 47months* | 37（1.9%) | 7（0.7%) | 30（3.0%) | 4.389（1.919，10.040） | <0.001 |
| BARC 1 or 2 bleeding events, n (%) |  |  |  |  |  |
| 24months | 103（5.2%) | 21（2.1%) | 82（8.3%) | 4.167（2.558，6.803） | <0.001 |
| 47months* | 174（8.8%) | 66（6.7%) | 108（11.0%) | 1.715（1.245，2.361） | 0.001 |

***：the median time of last follow-up was 47 months

*DAPT: dual antiplatelet therapy; OR: odds ratio; CI: confidence interval; BARC: bleeding academic research consortium.*
