# Supplementary material for: Prolonged dual antiplatelet therapy for Chinese ACS patients undergoing emergency PCI with drug-eluting stents: Benefits and risks
Source: Front Cardiovasc Med. 2023 Feb 9;10:1080673. doi: 10.3389/fcvm.2023.1080673 (PMC9976624; doi:10.3389/fcvm.2023.1080673)
Supplement: Supplementary file 4 [file Table_4.docx]

**Supplemental Table 4 Predictive value of DAPT duration for BARC 3 or 5 type bleeding events in Cox proportional hazard univariate and multivariate analysis**

| Characteristics | Univariate analysis | |  | Multivariate analysis | |
| --- | --- | --- | --- | --- | --- |
|  | HR (95%CI) | p value |  | HR（95%CI） | p value |
| Age | 1.032（1.001，1.064） | 0.041 |  |  |  |
| Hypertension | 1.859（0.979，3.532） | 0.058 |  |  |  |
| DM | 1.476（0.816，2.668） | 0.198 |  |  |  |
| DBP | 0.981（0.959，1.004） | 0.107 |  |  |  |
| SBP | 0.984（0.972，0.997） | 0.015 |  |  |  |
| Hb | 0.982（0.967，0.997） | 0.019 |  | 0.981（0.964，0.998） | 0.029 |
| LVEF | 0.981（0.957，1.005） | 0.121 |  |  |  |
| P2Y_12_ inhibitor | 1.189（0.469，3.012） | 0.716 |  | 1.377（0.534，3.546） | 0.508 |
| DAPT duration | 3.378（1.629，6.993） | 0.001 |  | 3.205（1.543，6.667） | 0.002 |

*DAPT: dual antiplatelet therapy; HR: hazard ratio; CI: confidence interval; DM: diabetes mellitus; DBP: diastolic blood pressure; SBP: systolic blood pressure; Hb: hemoglobin; LVEF: left ventricular ejection fracion.*
